# Factors Influencing Somatic Embryo Maturation in Sugi (Japanese Cedar, *Cryptomeria japonica* (Thunb. ex L.f.) D. Don)

**DOI:** 10.3390/plants10050874

**Published:** 2021-04-26

**Authors:** Tsuyoshi E. Maruyama, Saneyoshi Ueno, Hideki Mori, Takumi Kaneeda, Yoshinari Moriguchi

**Affiliations:** 1 Department of Research Planning and Coordination, Forestry and Forest Products Research Institute, Matsunosato 1, Tsukuba 305-8687, Japan; 2 Department of Forest Molecular Genetics and Biotechnology, Forestry and Forest Products Research Institute, Matsunosato 1, Tsukuba 305-8687, Japan; saueno@ffpri.affrc.go.jp (S.U.); morih@ffpri.affrc.go.jp (H.M.); 3 Graduate School of Science and Technology, Niigata University, Ikarashi 8050, Niigata 950-2181, Japan; takumi.kane01@gmail.com (T.K.); chimori@agr.niigata-u.ac.jp (Y.M.)

**Keywords:** ABA, amino acid, cotyledonary embryo, EM medium, KCl, PEG, somatic embryogenesis

## Abstract

This paper presents the results of several experiments identifying basal salts (BS) contained in maturation medium, polyethylene glycol (PEG) concentration, abscisic acid (ABA) concentration, additional supplementation with potassium chloride (KCl), amino acid (AA) concentration, and proliferation culture medium (PCM) as the main culture factors affecting somatic embryo maturation in sugi (Japanese cedar, *Cryptomeria japonica,* Cupressaceae). Highly efficient embryo maturation was achieved when embryogenic cell lines (ECLs) were cultured on media supplemented with a combination of PEG, ABA, and AAs. More than 1000 embryos per gram of fresh weight (FW) can be produced on EM maturation medium supplemented with 175 g L^−1^ PEG, 100 µM ABA, 2 g L^−1^ glutamine, 1 g L^−1^ asparagine, and 0.5 g L^−1^ arginine.

## 1. Introduction

Clonal propagation is an essential tool in biotechnology and breeding programs. Among the available clonal propagation methods, somatic embryogenesis (SE) is the most attractive technique for the large-scale propagation of conifers and long-term conservation of different embryogenic cell lines (ECLs) without changing their initial characteristics [1]. Additionally, SE is an ideal system for cell biology and molecular studies, including of the cell cycle, cell division, cell differentiation, and for understanding different genetic and epigenetic mechanisms for gene expression and phenotypic variation in plants. The mass propagation of conifers by SE, which was developed over the last three decades, has become the first biotechnology showing great potential for application in forestry [2]. However, for many species, in vitro clonal propagation is still difficult or inefficient [3]. The low efficiency of somatic embryo maturation is one of the major problems hampering efficient mass production and limiting practical applications [4]. Therefore, the high-quality somatic embryo production is one of the most important factors for using SE protocols in commercial propagation and breeding programs [5].

Sugi (Japanese Cedar, *Cryptomeria japonica* (Thunb. ex L.f.) D. Don, Cupressaceae), the most commercially important forest tree in Japan, is mainly used as construction wood, and today approximately 4.5 million ha (44% of the total artificial stands) are covered by sugi plantations [6]. Currently, breeding programs to produce elite trees (superior trees selected mainly on the basis of growth performance and wood quality) are being developed [7]. Furthermore, as a countermeasure against sugi pollinosis, a serious social and public health problem in Japan, efforts to produce superior male-sterile sugi trees are also being made [8]. In this context, the application of biotechnology to produce superior male-sterile trees in a short period of time is one of our main priorities. Now, efficient protocols to propagate male-sterile somatic plants of sugi involve combining the selection of embryogenic cells (ECs) with marker-assisted selection, and propagation through SE has been established [9].

Since SE was first reported for spruce species, numerous studies on conifer trees have been reported [10,11,12]. For sugi, after the first report on plant regeneration via SE [13], more results including studies on SE initiation [14,15,16,17,18], somatic embryo maturation [19,20,21,22], plant conversion [9,23], cryopreservation [24,25], and plant transformation [26,27] were published, showing progress in optimizing SE protocols. However, detailed reports on the factors influencing somatic embryo maturation in several ECLs from different seed families of sugi have not yet been published. Enhancing the high-quality embryo maturation efficiency is vitally important for improving SE protocols in mass production for practical uses. Against this background, this study aimed to obtain information from several ECLs analyzing the factors that affect the efficiency of somatic embryo maturation in *C. japonica*, to apply them to improving protocols for the production of somatic embryos. This paper describes the results of several experiments, including studies on the effect of basal salts (BS) contained in maturation medium, polyethylene glycol (PEG) concentration, abscisic acid (ABA) concentration, additional supplementation with potassium chloride (KCl), amino acid (AA) concentration, and proliferation culture medium (PCM), as the main culture factors affecting somatic embryo maturation in Japanese cedar.

## 2. Results and Discussion

### 2.1. Effect of Basal Salts (BS) in Medium on Somatic Embryo Maturation Efficiency

**Figure 1:**
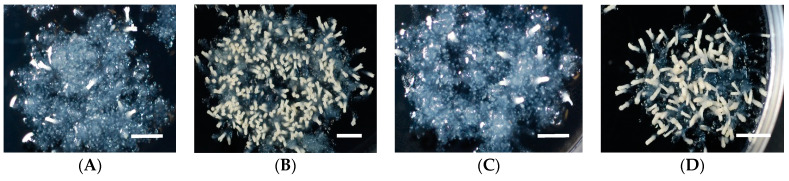
Somatic embryo maturation of sugi S-18 ECL on different media: (**A**) B5, (**B**) EM, (**C**) MS, and (**D**) WP media. Bars: 5 mm.

The first experimental approach used here was to determine the effect of BS contained in the medium on somatic embryo maturation efficiency in *C. japonica*. The mixtures of BS of three of the most popular media used in plant tissue culture were tested, comparing them with the BS of the EM medium [13] developed for SE of sugi. The results after eight weeks of culturing are shown in Table 1 and Figure 1. The best result was achieved using EM medium registering an average of 215.5 somatic embryos per plate, in comparison to 3.1, 14.8, and 48.8 embryos for MS, B5, and WP media, respectively. The highest maturation efficiency was achieved with S-18 ECL matured on EM medium (335.3 embryos) and the lowest efficiencies with S-73 and S-113 ECLs matured on MS medium (both 2.3 embryos). Although the statistical analysis results indicated that the number of embryos significantly differed depending on the BS of maturation media (deviance explained = 75.8%, df = 3, *p* < 0.001) (Table 2), statistically significant differences (*p* < 0.001) among ECLs were observed only within the WP maturation medium (Table S1). The unexpected results regarding to the low production of somatic embryos recorded using MS and B5 medium may be attributable to the high level of inorganic nitrogen (IN) present in both media. This would agree with the intermediate values obtained with the WP medium, which contains levels of IN intermediate between those of the high-content media (MS and B5 media) and the EM medium. A detailed study about the role of nitrogen during SE in Norway spruce was reported by Carlsson (2018) [28].

Similar to our experiment on *C. japonica*, the best results for embryo maturation in *Chamaecyparis pisifera* [29], *Larix decidua* [30], *Pinus nigra* [31], and *Picea abies* [32] were obtained in media supplemented with low IN, rather than the high concentration in the other media tested. In contrast, a survey of SE in crop plants noted that 70% of the explants were cultured on an MS medium or a modified version of it [33]. In conifers, several formulations of media are used; generally, the principal characteristic of these media is the reduction of NH_4_^+^ and NO_3_^-^ as IN from the standard concentrations and the addition of organic nitrogen sources, particularly L-glutamine. The addition of glutamine as a principal source of organic nitrogen to medium was reported in loblolly pine [34], radiata pine [35], Scots pine and maritime pine [36], Norway spruce [37,38], black spruce [39], and Fraser fir [40]. Notwithstanding the fact that numerous studies have reported that somatic embryos have been cultured in a range of media (from dilute formulations to high-concentration formulations), a suitable maturation medium for each species or ECL can only be developed by trial and error. However, traditionally, efforts to improve media based on empirical modifications of existing basic formulations can be inefficient and costly, and do not always produce the desired improvement [41]. Several media for coniferous plants have been developed with the aid of tissue analyses [42].

### 2.2. Effect of Polyethylene Glycol (PEG) Concentration in Medium on Somatic Embryo Maturation Efficiency

**Figure 2:**
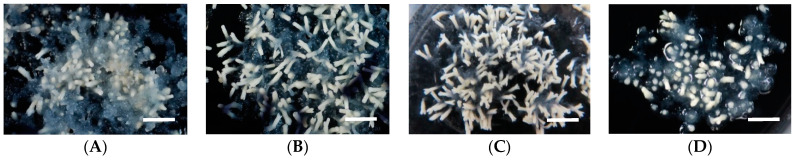
Somatic embryo maturation of sugi S-18 ECL on medium containing different PEG concentrations: (**A**) 10%, (**B**) 15%, (**C**) 17.5%, and (**D**) 20%. Bars: 5 mm.

The effect of PEG on embryo maturation efficiency was evaluated with five different ECLs of sugi. As shown in Table 3, for all of the lines tested, the number of embryos increased with increasing PEG concentration in the medium, reaching the highest peak at a concentration of 17.5% with maturation efficiency ranging from 446.3 to 812.7 embryos per plate. At a concentration of 20%, the number of achieved embryos dropped considerably to about one-fifteenth of the highest mean value. This result was consistent with our previous study on enhancing the maturation efficiency with increasing PEG concentrations, but inconsistent with the results obtained at a concentration of 20% as reported previously [22]. Differences in these results can be attributed to the differences in ECL genotypes and methodology used. In our present experiment, although a great difference in the phenotype of the mature embryos regarding to different PEG concentrations was not observed, active embryogenic cell proliferation was evident on medium containing 10% PEG, while the presence of many not well-developed embryos (short embryos) was frequently observed on medium supplemented with 20% PEG (Figure 2). Statistical analysis of our experimental results indicated that the maturation efficiency significantly differed among PEG concentrations in maturation media (deviance explained = 80.5%, df = 3, *p* < 0.001) (Table 4). No statistically significant differences were observed among the ECLs (Table S2).

PEG is one of the most popular osmoticants used in SE and reported to be an effective osmoregulator that plays an important role in enhancing embryo maturation efficiency in many conifers [5,11,29,43,44,45,46,47,48,49]. In this study, it was clearly demonstrated that, for *C. japonica*, the addition of PEG to the medium notably improved the embryo maturation efficiency. In the absence of PEG or at a low concentration of it, rarely or very low maturation frequency was observed, respectively (data not shown). This is consistent with the findings obtained in our previous studies where it was determined that increasing the osmolarity of the medium was necessary for the efficient production of somatic embryos [13,22].

### 2.3. Effect of Abscisic Acid (ABA) Concentration in Medium on Somatic Embryo Maturation Efficiency

ECs derived from seven different ECLs were used to test the effect of ABA concentration on embryo maturation efficiency in sugi. As shown in Table 5, the results among ABA treatments suggest that, with the exception of ECL S-85, which registered its best response at 200 µM, the appropriate concentration for embryo maturation is around 100 µM. The low number of cotyledonary embryos per plate achieved in the absence of ABA (6.5) was increased at 50 µM (201.4) and peaked at a concentration of 100 µM (336.2) but decreased when 200 µM (138.2) was added to the maturation medium. The number of mature embryos differed significantly among ABA concentrations of maturation medium (deviance explained = 71.9%, df = 3, *p < 0.001*) and there was also a significant interaction between ABA and ECLs (deviance explained = 12.4%, df = 3, *p <* 0.001) (Table 6, Table S3).

Numerous studies on the SE of conifers have indicated that ABA plays an important role in the efficient production of quality embryos, suggesting that ABA is closely related to the deposition of storage reserves (lipids, carbohydrates, and proteins) during the process of somatic embryo maturation, and prevents precocious germination [50,51,52]. Somatic embryos of spruces require higher levels of ABA to promote the normal development of plants [53,54]. Scots pine, maritime pine, radiata pine, and hybrid larches were produced normally with the addition of 60 µM ABA [36,55,56]. Conversely, ABA did not significantly benefit the somatic embryo maturation of some cypress trees, but also at a high concentration the cultures exhibited lower production, became necrotic, and did not undergo further development [57]. In addition, for some ECLs of European larch, the production of a large quantity of normal somatic embryos without the addition of ABA was reported [58]. However, for Japanese cedar, it was clearly demonstrated in this study that the number of mature embryos produced in the absence of ABA was far inferior, and only one of the seven ECLs tested was able to produce more than a dozen cotyledonary embryos per plate (Table 5). This response was similar to that of other Japanese conifers, in which the efficiency of embryo maturation was enhanced by the addition of 100 µM ABA to PEG-supplemented medium [5,29,45].

### 2.4. Effect of Additional Supplementation of Potasium Chloride (KCl) in Medium on Somatic Embryo Maturation Efficiency

The effect of the additional supplementation of KCl into medium on embryo maturation efficiency was evaluated with seven different ECLs of sugi. The results after eight weeks of culturing showed that the additional supplementation of KCl into maturation medium increased the number of cotyledonary embryos per plate in some ECLs (Table 7). However, the results of the data analysis indicated that the maturation efficiency did not differ significantly among the KCl concentrations and that there was no significant interaction of ECL:KCl supplementation (Table 8). With the exception of the ECL S-182, no statistically significant differences were observed among the ECLs (Table S4).

The addition of KCl to medium was reported to be beneficial for enhancing the induction of SE and maturation efficiency in loblolly pine [59,60]. Supplementation of KCl significantly enhanced initial-stage embryo formation and, when combined with 7.5% PEG, was reported to give the best result with the maximum number of mature embryos in loblolly pine [60]. However, the effect of KCl supplementation in enhancing embryo maturation in *C. japonica* was not statistically significant with the ECLs tested. The unclear effect of KCl supplementation can be attributed to the high concentration of PEG used in our experiment. Further study is needed to clarify the putative effect of KCl during the SE process in Japanese cedar.

### 2.5. Effect of Amino Acid (AA) Concentration in Medium on Somatic Embryo Maturation Efficiency

Table 9 shows the results on the effect of AA concentration in medium on somatic embryo maturation efficiency in five ECLs of sugi. The lowest result (11.3 embryos) achieved at AA 0× concentration indicates that the addition of AAs is essential for an efficient somatic embryo maturation in *C. japonica*. The best result was obtained in ECL S-18 at AA 2× concentration (452.7 embryos). Data analysis of our experimental results indicated that the maturation efficiency significantly differed among AA concentrations in maturation media (deviance explained = 70.6%, df = 3, *p* < 0.001), ECL (deviance explained = 6.8%, df = 4, *p* < 0.001), and depending on the interaction of ECL:AA concentration (deviance explained = 11.78%, df = 12, *p* < 0.001) (Table 10). However, these statistically significant differences among the variables were dependent on the lowest and highest efficiencies recorded by the AA 0× concentration and ECL S-18, respectively (Table S5).

**Figure 3:**
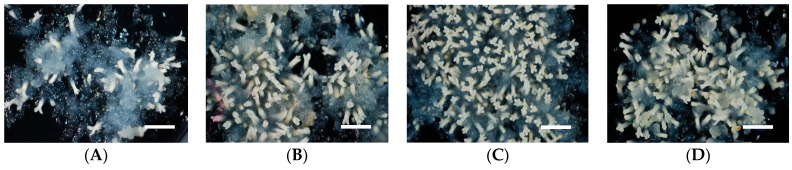
Somatic embryo maturation of sugi S-18 ECL on medium containing different amino acid (AA) concentrations: (**A**) 0×, (**B**) 1×, (**C**) 2×, and (**D**) 3×. Bars: 5 mm.

Organic nitrogen is essential for the production of healthy embryos during SE, and glutamine is one of the most common sources of organic nitrogen reported in conifers [32,61]. The addition of glutamine, asparagine, arginine, and other AAs to the maturation medium has been commonly used to enhance the maturation efficiency of somatic embryos [2,5,29,35,45,62,63,64,65,66,67]. The role of AAs during SE has been widely reported [28,40,68,69,70]. In our experiments, it was demonstrated that the addition of AAs was essential to obtain an efficient somatic embryo maturation in Japanese cedar. Although some ECL differences were observed regarding to the preferences for AA concentrations (i.e., S-113 preferred 1×, S-182 preferred 3×, and the other ECLs preferred 2×), supplementation of AA concentrations from 1× to 3× significantly improved the maturation efficiency (Table 9, Figure 3).

### 2.6. Effect of Proliferation Culture Medium (PCM) on Somatic Embryo Maturation Efficiency

As shown in Table 11, the average of somatic embryos varied from 318.9 to 384.6, but no statistically significant differences among the PCM were observed (Table 12). Data analysis of the GLM results indicated that the maturation efficiency significantly differed among ECLs (deviance explained = 27.2%, df = 3, *p* < 0.001), but the interaction of ECL:PCM was not significant (Table 12 and Table S6).

Although differences in the preferences for PCM were observed among ECLs (i.e., S-18 and T-158 preferred 3-1, S-100 preferred EM3-1, and T-1151 preferred EM3-1m) (Table 11) and all tested PCM were able to support the growth of ECs without significantly affecting the subsequent maturation process, the best somatic embryo maturation efficiency was achieved when ECLs were proliferated on 3-1 medium. This medium was also used as a maintenance-proliferation medium for other Japanese conifers [5,29,45,71,72,73,74,75]. It is well known that the composition of the medium significantly affects the initiation of SE as well as the efficiency of embryo maturation. In this context, recent improvements to the culture medium based on analytical studies of seed tissues, zygotic and somatic embryos, and gene expression in megagametophytes have been reported [12]. ECs of conifers are usually subcultured onto media of the same composition as those used for SE initiation or with some modification in terms of PGRs and sugar concentration. In some cases, weekly subcultures on PGR-free medium are recommended to minimize the effects of aging in ECs of maritime pine [76]. Similarly, subcultures of ECs on media with no PGRs were reported for radiata pine [35]. However, ECs of *C. japonica* subcultured on PGR-free medium led to embryo development and showed a decline in their proliferative capacity over time. The PCM developed for Japanese cedar supported the growth of ECLs by two- to three-week subculture routines for several years without losing their proliferative potential and initial morphological characteristics (data not shown).

### 2.7. Optimal Factors Giving the Best Results Achieved in Our Six Independent Experiments

The results of our six independent experiments with different ECLs, including studies on the effect of BS contained in maturation medium, PEG concentration, ABA concentration, additional supplementation of KCl, AA concentration, and PCM, as the main culture factors affecting somatic embryo maturation in sugi are summarized in Table 13.

## 3. Materials and Methods

### 3.1. Plant Material and Culture Conditions

Immature seeds collected from seed orchards were used as plant material for SE initiation. After isolation from collected cones, seeds were surface-sterilized with 1% (*w/v*) sodium hypochlorite solution for 15 min and then rinsed three times with sterile distilled water for 5 min each. Then, the entire megagametophytes were aseptically excised from seeds and used as explants. For the induction of ECs, explants were placed horizontally onto initiation medium contained in 90 × 15 mm quad-plates (three explants per well, 12 per plate) and cultured in the dark at 25 °C. Initiation medium containing BS (basal salts) reduced to half the concentration from the standard EM medium [13] was supplemented with 10 g L^−1^ sucrose, 10 μM 2,4-D, 5 μM BA, 0.5 g L^−1^ casein acid hydrolysate, 1 g L^−1^ glutamine, and solidified with 3 g L^−1^ gellan gum (Gelrite^®^; Wako Pure Chemical, Osaka, Japan). The pH was adjusted to 5.8 prior to autoclave the medium for 15 min at 121 °C.

### 3.2. Maintenance and Proliferation of Embryogenic Cells (ECs)

Induced ECs were subcultured every two to three weeks on maintenance/proliferation medium containing BS reduced to half the concentration from the standard EM medium [13] supplemented with 3 μM 2,4-D, 1 μM BA, 30 g L^−1^ sucrose, 1.5 g L^−1^ glutamine, and 3 g L^−1^ gellan gum. Clumps of ECs (12 per plate) were cultured in the dark at 25 °C. The ECs subcultured three to four times after induction were used for somatic embryo maturation.

### 3.3. Maturation of Somatic Embryos

#### 3.3.1. Effect of Basal Salts (BS) in Medium on Somatic Embryo Maturation Efficiency

For the maturation of somatic embryos, three-week-old proliferated ECs (early stage of somatic embryos characterized by an embryonal head with suspensor system) [13] from five ECLs (S-18, S-73, S-100, S-113, and S-352) were cultured in clumps (three masses per 90 × 20 mm plate, 100 mg each) on maturation medium for eight weeks. Maturation media containing the BS formulation of MS (Murashige and Skoog Medium M0237; Duchefa Biochemie B.V., Haarlem, the Netherlands), B5 (Gamborg’s B5 Medium Salt Mixture 399-00621; Wako Pure Chemical, Osaka, Japan), WP (McCown Woody Plant Medium M0220; Duchefa Biochemie B.V., Haarlem, the Netherlands), and EM medium [13] were supplemented with 30 g L^−1^ maltose, 2 g L^−1^ activated charcoal (AC), 100 µM abscisic acid (ABA), amino acids (AAs) (in g L^−1^: glutamine 2, asparagine 1, arginine 0.5, citrulline 0.079, ornithine 0.076, lysine 0.055, alanine 0.04, and proline 0.035), 175 g L^−1^ PEG (Av. Mol. Wt.: 7300–9300; Wako Pure Chemical, Osaka, Japan), and 3.3 g L^−1^ gellan gum (Table 1). The plates were sealed with Parafilm^®^ and kept in the dark at 25 °C. After eight weeks of culturing, the number of somatic embryos at the cotyledonary stage was recorded.

#### 3.3.2. Effect of Polyethylene Glycol (PEG) Concentration in Medium on Somatic Embryo Maturation Efficiency

To determine the effect of PEG concentration on the somatic embryo maturation of five ECLs (S-18, S-100, S-352, T-1151, and T-158), two-week-old proliferated ECs were cultured in clumps (five masses per 90 × 20 mm plate, 100 mg each) on maturation medium for eight weeks. Maturation medium containing the BS concentration of the standard EM medium [13] was supplemented with 30 g L^−1^ maltose, 2 g L^−1^ AC, 100 µM ABA, AAs (in g L^−1^: glutamine 2, asparagine 1, arginine 0.5, citrulline 0.079, ornithine 0.076, lysine 0.055, alanine 0.04, and proline 0.035), 3.3 g L^−1^ gellan gum, and 100, 150, 175, or 200 g L^−1^ PEG (Table 3). The plates were sealed with Parafilm^®^ and kept in the dark at 25 °C. After eight weeks of culturing, the number of somatic embryos at the cotyledonary stage was recorded.

#### 3.3.3. Effect of Abscisic Acid (ABA) Concentration in Medium on Somatic Embryo Maturation Efficiency

To determine the effect of ABA concentration on somatic embryo maturation, two-week-old proliferated cells from seven ECLs (S-18, S-64, S-85, S-100, S-113, S-352, and T-1151) were cultured in clumps (three masses per 90 × 20 mm plate, 100 mg each) on maturation medium for eight weeks. Maturation medium containing the BS concentration of the standard EM medium [13] was supplemented with 30 g L^−1^ maltose, 2 g L^−1^ AC, AAs (in g L^−1^: glutamine 2, asparagine 1, arginine 0.5, citrulline 0.079, ornithine 0.076, lysine 0.055, alanine 0.04, and proline 0.035), 175 g L^−1^ PEG, 3.3 g L^−1^ gellan gum, and 0, 50, 100, or 200 µM ABA (Table 5). The plates were sealed with Parafilm^®^ and kept in the dark at 25 °C. After eight weeks of culturing, the number of somatic embryos at the cotyledonary stage was recorded.

#### 3.3.4. Effect of Additional Supplementation of Potassium Chloride (KCl) to the Medium on Somatic Embryo Maturation Efficiency

The effect of additional supplementation of KCl to the maturation medium was evaluated with seven ECLs (S-18, S-85, S-100, S-182, S-352, T-1151, and T-158). Two-week-old proliferated ECs were cultured in clumps (three masses per 90 × 20 mm plate, 100 mg each) on maturation medium for eight weeks. Maturation medium containing the BS concentration of the standard EM medium [13] was supplemented with 30 g L^−1^ maltose, 2 g L^−1^ AC, 100 µM ABA, AAs (in g L^−1^: glutamine 2, asparagine 1, arginine 0.5, citrulline 0.079, ornithine 0.076, lysine 0.055, alanine 0.04, and proline 0.035), 175 g L^−1^ PEG, 3.3 g L^−1^ gellan gum, and with (+) or without (–) additional supplementation of 0.67 g L^−1^ (9 mM) KCl (Table 7). The KCl concentration in medium without (–) and with (+) additional supplementation was 0.08 g L^−1^ (1 mM) and 0.75 g L^−1^ (10 mM), respectively. The plates were sealed with Parafilm^®^ and kept in the dark at 25 °C. After eight weeks of culturing, the number of somatic embryos at the cotyledonary stage was recorded.

#### 3.3.5. Effect of Amino Acid (AA) Concentration in Medium on Somatic Embryo Maturation Efficiency

The effect of AA concentration in maturation medium was evaluated with five ECLs (S-18, S-100, S-113, S-182, and S-352). Two-week-old proliferated ECs were cultured in clumps (three masses per 90 × 20 mm plate, 100 mg each) on maturation medium for eight weeks. Maturation medium containing the BS concentration of the standard EM medium [13] was supplemented with 30 g L^−1^ maltose, 2 g L^−1^ AC, 100 µM ABA, 175 g L^−1^ PEG, 3.3 g L^−1^ gellan gum, and AAs (in g L^−1^: glutamine 0–3, asparagine 0–1.5, arginine 0–0.75, citrulline 0.079, ornithine 0.076, lysine 0.055, alanine 0.04, and proline 0.035). The AA concentration 1× represents the addition of 1 g L^−1^ glutamine, 0.5 g L^−1^ asparagine, and 0.25 g L^−1^ arginine (major AA mix). The AA concentrations 2× and 3× represent the addition of two and three times the concentration of the major AA mix of 1×, respectively. Minor AAs (citrulline, ornithine, lysine, alanine, and proline) were added at the same concentrations as described above for 1×, 2×, and 3×. The AA concentration 0× represents no addition of AAs to the medium (Table 9). The plates were sealed with Parafilm^®^ and kept in the dark at 25 °C. After eight weeks of culturing, the number of somatic embryos at the cotyledonary stage was recorded.

#### 3.3.6. Effect of Proliferation Culture Medium (PCM) on Somatic Embryo Maturation Efficiency

The effect of PCM on somatic embryo maturation was evaluated with four ECLs (S-18, S-100, T-1151, and T-158). The following media, named 3-1, EM3-1, EM3-1m, and EM10-0, were tested as PCM of ECs before maturation (Table 6). The composition of 3-1 medium was the same as described above in the section on the maintenance and proliferation of ECs. The EM3-1 medium represents the formulation of 3-1 medium but containing BS at the standard concentration of EM medium [13]. The EM3-1m medium had the same formulation as the EM3-1 medium but contained maltose instead of sucrose at the same concentration. The EM10-0 medium had the same formulation as EM3-1 medium but contained 10 μM 2,4-D and no BA. Subsequently, after two weeks of culture on different PCM, ECs were cultured in clumps (three masses per 90 × 20 mm plate, 100 mg each) on maturation medium for eight weeks. Maturation medium containing the BS concentration of the standard EM medium [13] was supplemented with 30 g L^−1^ maltose, 2 g L^−1^ AC, 100 µM ABA, AAs (in g L^−1^: glutamine 2, asparagine 1, arginine 0.5, citrulline 0.079, ornithine 0.076, lysine 0.055, alanine 0.04, and proline 0.035), 175 g L^−1^ PEG, and 3.3 g L^−1^ gellan gum. The plates were sealed with Parafilm^®^ and kept in the dark at 25 °C. After eight weeks of culturing, the number of somatic embryos at the cotyledonary stage was recorded.

### 3.4. Data Analysis

The effects of the six types of trial (BS, PEG, ABA, KCl, AA, and PCM), ECLs, interactions between the trials and the ECLs, and the replicates (plates) on somatic embryo maturation in sugi were analyzed using negative binomial generalized linear models (GLMs). To compare the importance of each variable, percent deviance explained (%) was calculated as {1– (residual deviance) / (null deviance)} × 100, where residual deviance is the deviance of each variable, and null deviance is the deviance of the null model (i.e., intercept only model). The post hoc analysis of Tukey’s all-pair comparisons for the GLMs was carried out to evaluate significant differentiation within the trials and the ECLs. The GLMs were fitted using R package “MASS” [77], and the multiple comparisons within the trials and the ECLs were performed using R package “multcomp” [78].

## 4. Concluding Remarks

Genotypes, BS formulations, sugars, AAs, PGRs, osmotic agents, and culture conditions are some of the principal factors controlling SE [10,79,80,81,82,83,84,85,86,87]. Highly efficient embryo maturation was achieved when ECLs were cultured on media supplemented with a combination of PEG, ABA, and AAs. More than 1000 embryos per gram (FW) can be produced on media supplemented with 175 g L^−1^ PEG, 100 µM ABA, and 2× AA concentration. This result represents a significant improvement in somatic embryo maturation efficiency compared with that in our previous studies [13,22]. Although the results obtained in this study analyzing the main factors affecting the efficiency of somatic embryo maturation in *C. japonica* could be used to enhance the protocols for producing high-quality somatic embryos, it is necessary to consider that, given that SE is a complex multistage process influenced by the active interaction of several variables, the results obtained in our independent experiments may vary with the interaction of the factors. Despite the fact that more efforts, including studies on the interaction of main factors, are necessary to maximize the efficiency in the production of somatic embryos for practical purposes, to our knowledge, this is the first detailed report on the main factors affecting somatic embryo maturation in Japanese cedar, which should contribute to improving SE protocols. In addition, we believe that this report can also provide useful information to improve embryo maturation efficiency in other conifers.

## Figures and Tables

**Table 1:** Effect of basal salts (BS) of medium on somatic embryo maturation efficiency of sugi embryogenic cell lines (ECLs). Data are presented as the mean ± SD (standard deviation) of the number of cotyledonary somatic embryos per plate from three replicates for each ECL matured on media containing different BS formulations.

ECL	Cotyledonary Somatic Embryos per Plate by BS Formulations of Maturation Medium				
	B5	EM	MS	WP	Average per Cell Line
**S-18**	34.7 ± 7.4	335.3 ± 136.1	5.0 ± 1.0	134.7 ± 20.0	127.4 ± 147.3 a
**S-73**	7.7 ± 5.0	223.0 ± 56.2	2.3 ± 1.5	87.3 ± 39.7	80.1 ± 97.6 ab
**S-100**	11.0 ± 9.0	235.0 ± 145.2	2.7 ± 2.5	6.3 ± 5.9	63.8 ± 120.5 ab
**S-113**	9.3 ± 11.0	130.7 ± 39.0	2.3 ± 0.6	5.7 ± 4.0	37.0 ± 59.2 b
**S-352**	11.3 ± 9.5	153.3 ± 83.7	3.0 ± 1.0	10.0 ± 7.8	44.4 ± 75.0 ab
**Average per medium**	14.8 ± 12.7 c	215.5 ± 113.4 a	3.1 ± 1.6 d	48.8 ± 57.5 b	

Different letters show significant differences according to Tukey’s post hoc test (*p* < 0.05).

**Table 2:** Summary of deviances of the generalized linear model (GLM) of data from Table 1 (Effect of basal salts (BS) of medium on somatic embryo maturation efficiency of sugi embryogenic cell lines (ECLs)). Deviance of the variable “null” indicates the null deviance (deviance of the null model), and other deviances indicate the residual deviance (deviance of the variable).

Variables	df	Residual df	Deviances	*p* Value	Significance	Deviance Explained (%)
Null		59	925.01			
ECL	4	55	76.45	<0.001	***	8.3
BS	3	52	701.02	<0.001	***	75.8
Plate	2	50	6.02	<0.05	*	0.7
ECL:BS	12	38	76.14	<0.001	***	8.2

**Table 3:** Effect of polyethylene glycol (PEG) concentration on somatic embryo maturation efficiency of sugi embryogenic cell lines (ECLs). Data are presented as the mean ± SD (standard deviation) of the number of cotyledonary somatic embryos per plate from three replicates for each ECL matured on media containing different concentrations of PEG.

ECL	Cotyledonary Somatic Embryos per Plate by PEG Concentrations				
	PEG 10%	PEG 15%	PEG 17.5%	PEG 20%	Average per Cell Line
**S-18**	222.3 ± 95.6	544.0 ± 200.3	640.3 ± 61.0	36.7 ± 7.8	360.8 ± 272.0 a
**S-100**	160.3 ± 45.6	322.3 ± 109.3	532.0 ± 120.0	20.0 ± 17.8	258.7 ± 211.8 a
**S-352**	210.0 ± 100.5	395.7 ± 209.9	446.3 ± 145.4	51.7 ± 19.6	275.9 ± 201.2 a
**T-1151**	222.3 ± 127.6	461.3 ± 77.6	812.7 ± 196.2	58.7 ± 18.2	388.8 ± 314.4 a
**T-158**	157.7 ± 73.0	317.0 ± 106.2	686.0 ± 172.9	43.7 ± 25.8	301.1 ± 269.7 a
**Average per PEG** **concentration**	194.5 ± 84.0 b	408.1 ± 155.4 a	623.5 ± 180.2 a	42.1 ± 21.0 c	

Different letters show significant differences according to Tukey’s post hoc test (*p* < 0.05).

**Table 4:** Summary of deviances of the generalized linear model (GLM) of data from Table 3 (Effect of polyethylene glycol (PEG) concentration on somatic embryo maturation efficiency of sugi embryogenic cell lines (ECLs)). Deviance of the variable “null” indicates the null deviance (deviance of the null model), and other deviances indicate the residual deviance (deviance of the variable).

Variables	df	Residual df	Deviances	*p* Value	Significance	Deviance Explained (%)
Null		59	515.742			
ECL	4	55	13.13289	< 0.05	*	2.5
PEG concentration	3	52	415.1126	< 0.001	***	80.5
Plate	2	50	0.261557	0.877	ns	0.1
ECL:PEG concentration	12	38	14.18647	0.289	ns	2.8

ns, not significant.

**Table 5:** Effect of abscisic acid (ABA) concentration on somatic embryo maturation efficiency of sugi embryogenic cell lines (ECLs). Data are presented as the mean ± SD (standard deviation) of the number of cotyledonary somatic embryos per plate from three replicates for each ECL matured on media containing different concentrations of ABA.

ECL	Cotyledonary Somatic Embryos per Plate by ABA Concentrations				
	ABA 0 µM	ABA 50 µM	ABA 100 µM	ABA 200 µM	Average per Cell Line
**S-18**	19.3 ± 10.5	186.3 ± 161.1	385.0 ± 117.4	163.7 ± 49.9	188.6 ± 161.9 a
**S-64**	2.0 ± 1.7	273.0 ± 206.9	366.0 ± 128.3	22.3 ± 8.6	165.8 ± 194.4 b
**S-85**	2.3 ± 2.5	237.7 ± 18.0	256.7 ± 64.0	344.7 ± 129.6	210.3 ± 146.2 b
**S-100**	8.7 ± 7.8	354.0 ± 62.4	377.3 ± 119.4	166.0 ± 117.5	226.5 ± 174.4 ab
**S-113**	1.7 ± 2.1	37.3 ± 18.9	228.7 ± 113.1	77.7 ± 12.0	86.3 ± 102.8 b
**S-352**	3.3 ± 0.6	50.0 ± 25.5	331.7 ± 59.5	54.3 ± 27.2	109.8 ± 138.7 b
**T-1151**	8.0 ± 4.6	271.7 ± 98.3	408.3 ± 205.9	139.0 ± 13.5	206.8 ± 183.7 ab
**Average per ABA** **concentration**	6.5 ± 7.5 c	201.4 ± 145.2 ab	336.2 ± 122.3 a	138.2 ± 116.8 b	

Different letters show significant differences according to Tukey’s post hoc test (*p* < 0.05).

**Table 6:** Summary of deviances of the generalized linear model (GLM) of data from Table 5 (Effect of abscisic acid (ABA) concentration on somatic embryo maturation efficiency of sugi embryogenic cell lines (ECLs)). Deviance of the variable “null” indicates the null deviance (deviance of the null model), and other deviances indicate the residual deviance (deviance of the variable).

Variables	df	Residual df	Deviances	*p* Value	Significance	Deviance Explained (%)
Null		83	1078.277			
ECL	6	77	66.60139	<0.001	***	6.2
ABA concentration	3	74	775.4969	<0.001	***	71.9
Plate	2	72	6.15035	<0.05	*	0.6
ECL:ABA concentration	18	54	134.1964	<0.001	***	12.4

**Table 7:** Effect of potassium chloride (KCl) supplementation on somatic embryo maturation efficiency of sugi embryogenic cell lines (ECLs). Data are present as the mean ± SD (standard deviation) of the number of cotyledonary somatic embryos per plate from five replicates for each ECL matured on medium with (+) or without (–) additional KCl supplementation.

ECL	Cotyledonary Somatic Embryos per Plate by Additional KCl Supplementation		
	KCl (–)	KCl (+)	Average per Cell Line
**S-18**	381.0 ± 151.0	420.8 ± 72.4	400.9 ± 113.6 ab
**S-85**	294.4 ± 111.7	214.4 ± 43.0	254.4 ± 90.2 ab
**S-100**	325.2 ± 73.3	309.2 ± 68.7	317.2 ± 67.5 ab
**S-182**	197.4 ± 62.7	208.2 ± 91.0	202.8 ± 73.9 b
**S-352**	304.2 ± 204.7	336.2 ± 105.0	320.2 ± 154.3 ab
**T-1151**	459.8 ± 289.8	362.6 ± 263.8	411.2 ± 266.2 a
**T-158**	385.4 ± 126.6	391.8 ± 144.8	388.6 ± 128.3 ab
**Average per KCl supplementation**	335.3 ± 167.6 a	320.5 ± 142.7 a	

Different letters show significant differences according to Tukey’s post hoc test (*p* < 0.05).

**Table 8:** Summary of deviances of the generalized linear model (GLM) of data from Table 7 (Effect of potassium chloride (KCl) supplementation on somatic embryo maturation efficiency of sugi embryogenic cell lines (ECLs)). Deviance of the variable “null” indicates the null deviance (deviance of the null model), and other deviances indicate the residual deviance (deviance of the variable).

Variables	df	Residual df	Deviances	*p* Value	Significance	Deviance Explained (%)
Null		69	100.8604			
ECL	6	63	25.15085	<0.001	***	24.9
KCl supplementation	1	62	0.257053	0.612	ns	0.3
Plate	4	58	0.92416	0.921	ns	0.9
ECL:KCl supplementation	6	52	2.775315	0.836	ns	2.8

ns, not significant.

**Table 9:** Effect of amino acid (AA) concentration on somatic embryo maturation efficiency of sugi embryogenic cell lines (ECLs). Data are presented as the mean ± SD (standard deviation) of the number of cotyledonary somatic embryos per plate from three replicates for each ECL matured on media containing different concentrations of AAs.

ECL	Cotyledonary Somatic Embryos per Plate by AA Concentrations				
	0×	1×	2×	3×	Average per Cell Line
**S-18**	42.0 ± 33.4	330.3 ± 211.8	452.7 ± 221.2	305.0 ± 155.9	282.5 ± 214.7 a
**S-100**	3.0 ± 2.6	156.3 ± 74.9	340.7 ± 53.6	274.7 ± 39.5	193.7 ± 140.7 b
**S-113**	6.7 ± 2.5	208.7 ± 69.2	115.7 ± 38.6	94.3 ± 72.2	106.3 ± 87.9 b
**S-182**	2.3 ± 2.1	233.7 ± 83.3	274.0 ± 75.6	308.0 ± 152.6	204.5 ± 148.8 b
**S-352**	2.3 ± 2.5	268.7 ± 73.7	353.3 ± 50.5	302.7 ± 110.7	231.8 ± 154.3 b
**Average per AA** **concentration**	11.3 ± 20.5 a	239.5 ± 115.3 b	307.3 ± 148.7 b	256.9 ± 129.4 b	

Different letters show significant differences according to Tukey’s post hoc test (*p* < 0.05).

**Table 10:** Summary of deviances of the generalized linear model (GLM) of data from Table 9 (Effect of amino acid (AA) concentration on somatic embryo maturation efficiency of sugi embryogenic cell lines (ECLs)). Deviance of the variable “null” indicates the null deviance (deviance of the null model), and other deviances indicate the residual deviance (deviance of the variable).

Variables	df	Residual df	Deviances	*p* Value	Significance	Deviance Explained (%)
Null		59	627.6505			
ECL	4	55	42.45872	<0.001	***	6.8
AA concentration	3	52	442.8232	<0.001	***	70.6
Plate	2	50	0.919119	0.632	ns	0.1
ECL:AA concentration	12	38	73.40887	<0.001	***	11.7

ns, not significant.

**Table 11:** Effect of proliferation culture media (PCM) on somatic embryo maturation efficiency of sugi embryogenic cell lines (ECLs). Data are presented as the mean ± SD (standard deviation) of the number of cotyledonary somatic embryos per plate from five replicates for each ECL matured after culturing on different PCM.

ECL	Cotyledonary Somatic Embryos per Plate by Proliferation Media				
	3-1	EM3-1	EM3-1m	EM10-0	Average per Cell line
**S-18**	381.6 ± 88.7	345.2 ± 133.5	269.6 ± 48.4	274.2 ± 143.9	317.6 ± 112.4 ab
**S-100**	268.0 ± 80.3	284.0 ± 70.7	233.6 ± 69.3	252.0 ± 37.7	259.4 ± 63.9 b
**T-1151**	478.8 ± 115.0	458. 4± 89.8	493.6 ± 100.1	450.0 ± 130.5	470.2 ± 102.4 a
**T-158**	410.0 ± 293.7	402.2 ± 256.8	351.4 ± 114.6	299.4 ± 94.7	365.8 ± 196.9 ab
**Average per PCM**	384.6 ± 173.3 a	372.4 ± 157.6 a	337.1 ± 130.0 a	318.9 ± 128.3 a	

Different letters show significant differences according to Tukey’s post hoc test (*p* < 0.05).

**Table 12:** Summary of deviances of the generalized linear model (GLM) of data from Table 11 (effect of proliferation culture media (PCM) on somatic embryo maturation efficiency of sugi embryogenic cell lines (ECLs)). Deviance of the variable “null” indicates the null deviance (deviance of the null model), and other deviances indicate the residual deviance (deviance of the variable).

Variables	df	Residual df	Deviances	*p* Value	Significance	Deviance Explained (%)
Null		79	126.5727			
ECL	3	76	34.46032	<0.001	***	27.2
PCM	3	73	4.761264	0.190	ns	3.8
Plate	4	69	2.787561	0.594	ns	2.2
ECL:PCM	9	60	3.155583	0.958	ns	2.5

ns, not significant.

**Table 13:** Summary of the optimal factors giving the best results achieved in our six independent experiments including studies on the effect of basal salts (BS) contained in maturation medium, polyethylene glycol (PEG) concentration, abscisic acid (ABA) concentration, additional supplementation of potassium chloride (KCl), amino acid (AA) concentration, and proliferation culture media (PCM), as the main culture factors affecting somatic embryo maturation in sugi.

Factor	Best Result	Reference
BS in medium	EM	Maruyama et al. 2000
PEG concentration	175 g L^−1^	169-09125 Wako Pure Chemical, Osaka, Japan
ABA concentration	100 µM	BIA-0125 Apollo Scientific Ltd., Manchester, UK
KCl concentration	0.08 g L^−1^	063-03545 Wako Pure Chemical, Osaka, Japan
AA concentration	L (+) Glutamine 2 g L^−1^ L- Asparagine 1 g L^−1^ L (+) Arginine 0.5 g L^−1^	078-00525 Wako Pure Chemical, Osaka, Japan 013-04815 Wako Pure Chemical, Osaka, Japan 011-04615 Wako Pure Chemical, Osaka, Japan
PCM	3-1 (EM proliferation medium)	Maruyama et al. 2000

## Supplementary Materials

The following are available online at https://www.mdpi.com/article/10.3390/plants10050874/s1, Supplementary Table S1: Summary of the estimated parameters of the generalized linear model (GLM) of data from Table 1 (Effect of basal salts (BS) of medium on somatic embryo maturation efficiency of sugi embryogenic cell lines (ECLs)). Supplementary Table S2: Summary of the estimated parameters of the generalized linear model (GLM) of data from Table 3 (Effect of polyethylene glycol (PEG) concentration on somatic embryo maturation efficiency of sugi embryogenic cell lines (ECLs)). Supplementary Table S3: Summary of the estimated parameters of the generalized linear model (GLM) of data from Table 5 (Effect of abscisic acid (ABA) concentration on somatic embryo maturation efficiency of sugi embryogenic cell lines (ECLs)). Supplementary Table S4: Summary of the estimated parameters of the generalized linear model (GLM) of data from Table 7 (Effect of potassium chloride (KCl) supplementation on somatic embryo maturation efficiency of sugi embryogenic cell lines (ECLs)). Supplementary Table S5: Summary of the estimated parameters of the generalized linear model (GLM) of data from Table 9 (Effect of amino acid (AA) concentration on somatic embryo maturation efficiency of sugi embryogenic cell lines (ECLs)). Supplementary Table S6: Summary of the estimated parameters of the generalized linear model (GLM) of data from Table 11 (Effect of proliferation culture media (PCM) on somatic embryo maturation efficiency of sugi embryogenic cell lines (ECLs)).

## References

[1] Park J.S., Barret J.D., Bonga J.M. (1998). Application of somatic embryogenesis in high-value clonal forestry: Deployment, genetic control, and stability of cryopreserves clones. Cell. Dev. Biol. Plant.

[2] Klimaszewska K., Trontin J.F., Becwar M.R., Devillard C., Park Y.S., Lelu-Walter M.A. (2007). Recent progress in somatic embryogenesis of four *Pinus* spp.. Tree For. Sci. Biotechol..

[3] Bonga J.M., Klimaszewska K., von Aderkas P. (2010). Recalcitrance in clonal propagation, in particular of conifers. Plant Cell Tissue Organ Cult..

[4] Maruyama E.T., Hosoi Y. (2012). Post-maturation treatments improves and synchronizes somatic embryo germination of three species of Japanese pines. Plant Cell Tissue Organ Cult..

[5] Maruyama E.T., Hosoi Y. (2019). Progress in somatic embryogenesis of Japanese pines. Front. Plant Sci..

[6] Forestry Agency (2014). Statistical Handbook of Forest and Forestry.

[7] Saito M., Teranishi H. (2014). A breeding strategy of male sterile *Cryptomeria japonica* D. Don cultivars. Jpn. J. Palynol..

[8] Moriguchi Y., Ueno S., Higuchi Y., Miyajima D., Itoo S., Futamura N., Shinohara K., Tsumura Y. (2014). Establishment of a microsatellite panel covering the sugi (*Cryptomeria japonica*) genome, and its application for localization of a male-sterile gene (*ms-2*). Mol. Breed..

[9] Maruyama E.T., Ueno S., Hirayama S., Kaneeda T., Moriguchi Y. (2020). Somatic embryogenesis and plant regeneration from sugi (Japanese Cedar, *Cryptomeria japonica* D. Don, Cupressaceae) seed families by marker assisted selection for the male sterility allele *ms1*. Plants.

[10] Tautorus T.E., Fowke L.C., Dunstan D.I. (1991). Somatic embryogenesis in conifers. Can. J. Bot..

[11] Jain S.M., Gupta P.K., Newton R.J. (1995). Somatic Embryogenesis in Woody Plants.

[12] Klimaszewska K., Hargreaves C., Lelu-Walter M.A., Trontin J.F., Germana M., Lambardi M. (2016). Advances in conifer somatic embryogenesis since year 2000. In Vitro Embryogenesis in Higher Plants, Methods in Molecular Biology.

[13] Maruyama E., Tanaka T., Hosoi Y., Ishii K., Morohoshi N. (2000). Embryogenic cell culture, protoplast regeneration, cryopreservation, biolistic gene transfer and plant regeneration in Japanese cedar (*Cryptomeria japonica* D. Don). Plant Biotechnol..

[14] Maruyama E.T., Hosoi Y., Futamura N., Saito M. (2014). Initiation of embryogenic cultures from immature seeds of pollen-free sugi (*Cryptomeria japonica*). Kanto Shinrin Kenkyu.

[15] Maruyama E.T., Hosoi Y., Ueno S., Onishi N., Onishi N., Totsuka S., Iwai J., Moriguchi Y. (2017). Somatic embryogenic cell induction from seed of pollen-free sugi (*Cryptomeria japonica*) produced at the Niigata prefecture. Kanto Shinrin Kenkyu.

[16] Maruyama E.T., Miyazawa S., Ueno S., Onishi N., Totsuka S., Iwai J., Moriguchi Y. (2018). Differences among families on embryogenic cell induction from seed of pollen-free sugi (*Cryptomeria japonica*) produced at the Niigata prefecture. Kanto Shinrin Kenkyu.

[17] Maruyama E.T., Kaneeda T., Ueno S., Hirayama S., Itoh Y., Bamba Y., Moriguchi Y. (2020). Embryogenic cell induction from immature seeds derived from polycross of sugi (*Cryptomeria japonica*). Kanto Shinrin Kenkyu.

[18] Maruyama E.T., Ueno S., Hosoi Y., Miyazawa S.-I., Mori H., Kaneeda T., Bamba Y., Itoh Y., Hirayama S., Kawakami K. (2021). Somatic embryogenesis initiation in sugi (Japanese Cedar, *Cryptomeria japonica* D. Don): Responses from male-fertile, male-sterile, and polycoss-pollinated-derived seed explants. Plants.

[19] Igasaki T., Sato T., Akashi N., Mohri T., Maruyama E., Kinoshita I., Walter C., Shinohara K. (2003). Somatic embryogenesis and plant regeneration from immature zygotic embryos of *Cryptomeria japonica* D. Don. Plant Cell Rep..

[20] Igasaki T., Akashi N., Ujino-Ihara T., Matsubayashi Y., Sakagami Y., Shinohara K. (2003). Phytosulfokine stimulates somatic embryogenesis in *Cryptomeria japonica*. Plant Cell Physiol..

[21] Nakagawa R., Ogita S., Kubo T., Funada R. (2006). Effect of polyamines and L-ornithine on the development of proembryogenic masses of *Cryptomeria japonica*. Plant Cell Tissue Organ Cult..

[22] Maruyama E., Hosoi Y. (2007). Polyethylene glycol enhance somatic embryo production in Japanese cedar (*Cryptomeria japonica* D. Don). Propag. Ornam. Plants.

[23] Maruyama E.T., Hosoi Y., Miyazawa S., Ueno S., Onishi N., Totsuka S., Iwai J., Moriguchi Y. (2019). Pollen-free plant regeneration from embryogenic cells derived from sugi (*Cryptomeria japonica*). Kanto Shinrin Kenkyu.

[24] Maruyama E.T., Ishii K., Hosoi Y., Takao N., Hirai D., Matsumoto T., Tanaka D. (2016). Cryopreservation of embryogenic cells of sugi (*Cryptomeria japonica*). Cryopreservation of Plant Cells and Organs.

[25] Taniguchi T., Konagaya K., Nanasato Y. (2020). Somatic embryogenesis in artificially pollinated seed families of 2nd generation plus trees and cryopreservation of embryogenic tissue in *Cryptomeria japonica* D. Don (sugi). Plant Biotechnol..

[26] Taniguchi T., Ohmiya Y., Kurita M., Tsubomura M., Kondo T. (2008). Regeneration of transgenic *Cryptomeria japonica* D. Don after *Agrobacterium tumefaciens*-mediated transformation of embryogenic tissue. Plant Cell Rep..

[27] Konagaya K., Kurita M., Taniguchi T. (2013). High-efficiency *Agrobacterium*-mediated transformation of *Cryptomeria japonica* D. Don by co-cultivation on filter paper wicks followed by meropenem treatment to eliminate *Agrobacterium*. Plant Biotechnol..

[28] Carlsson J. (2018). Nitrogen Uptake and Assimilation during Norway Spruce Somatic Embryogenesis: Investigating the Role of Glutamine. Doctoral Thesis.

[29] Maruyama E., Hosoi Y., Ishii K. (2002). Somatic embryogenesis in Sawara cypress (*Chamaecyparis pisifera* Sieb. et Zucc.) for stable and efficient plant regeneration, propagation and protoplast culture. J. For. Res..

[30] Bonga J.M. (2004). The effect of various culture media on the formation of embryo-like structures in cultures derived from explants taken from mature *Larix decidua*. Plant Cell Tissue Organ Cult..

[31] Salaj T., Fráterová L., Cárach M., Salaj J. (2014). The effect of culture medium formulation on *Pinus nigra* somatic embryogenesis. Dendrobiology.

[32] Dahrendorf J., Clapham D., Egertsdotter U. (2018). Analisys of nitrogen utilization capability during the proliferation and maturation phases of Norway spruce (*Picea abies* (L.) H. Karst) somatic embryogenesis. Forests.

[33] Ammirato P.V., Evans D.A., Sharp W.R., Ammirato P.V., Yamada Y. (1983). Embryogenesis. Hand Book of Plant Cell Culture, Techniques for Propagation and Breeding.

[34] Becwar M.R., Nagmani R., Wann S.R. (1990). Initiation of embryogenic cultures and somatic embryo development in loblolly pine (*Pinus taeda*). Can. J. For. Res..

[35] Smith D.R. (1996). Growth Medium. U.S. Patent.

[36] Lelu M.A., Bastien C., Drugeault A., Gouez M.L., Klimaszewska K. (1999). Somatic embryogenesis and plantlet development in *Pinus sylvestris* and *Pinus pinaster* on medium with and without growth regulators. Physiol. Plant..

[37] Bozhkov P.V., Mikhlina S.B., Shiryaeva G.A., Lebedenko L.A. (1993). Influence of nitrogen balance of culture medium on Norway spruce (*Picea abies* (L.) Karst) somatic polyembryogenesis: High frequency establishment of embryonal-suspensor mass lines from mature zygotic embryos. J. Plant. Physiol..

[38] Carlsson J., Svennerstam H., Moritz T., Egertsdotter U., Ganeteg U. (2017). Nitrogen uptake and assimilation in proliferating embryogenic cultures of Norway spruce–Investigating the specific role of glutamine. PLoS ONE.

[39] Khlifi S., Tremblay F. (1995). Maturation of black spruce somatic embryos. Part I. Effect of L-glutamine on the number and germinability of somatic embryos. Plant Cell Tissue Organ Cult..

[40] Pullman G., Olson K., Fischer T., Egertsdotter U., Frampton J., Bucalo K. (2016). Fraser fir somatic embryogenesis: High frequency initiation, maintenance, embryo development, germination and cryopreservation. New For..

[41] Pullman G.S., Zeng H., Copeland-Kamp B., Crockett J., Lucrezi J., May S.W., Bucalo K. (2015). Conifer somatic embryogenesis: Improvements by supplementation of medium with oxidation-reduction agents. Tree Physiol..

[42] Pullman G.S., Bucalo K. (2014). Pine somatic embryogenesis: Analyses of seed tissue and medium to improve protocol development. New For..

[43] Li X.Y., Huang F.H., Murphy J.B., Gbur E.E. (1998). Polyethylene glycol and maltose enhance somatic embryo maturation in loblolly pine (*Pinus taeda* L.). Cell. Dev. Biol. Plant.

[44] Salaj T., Matúŝová R., Salaj J. (2004). The effect of carbohydrates and polyethylene glycol on somatic embryo maturation in hybrid fir *Abies alba* x *Abies numidica*. Acta Biol. Crac..

[45] Maruyama E., Ishii K., Hosoi Y. (2005). Efficient plant regeneration of Hinoki cypress (*Chamaecyparis obtusa* Sieb. et Zucc.) via somatic embryogenesis. J. For. Res..

[46] Krajñáková J., Häggman H., Gömöry D. (2009). Effect of sucrose concentration, polyethylene glycol and activated charcoal on maturation and regeneration of *Abies cephalonica* somatic embryos. Plant Cell Tissue Organ Cult..

[47] Shoji M., Sato H., Nakagawa R., Funada R., Kubo T., Ogita S. (2006). Influence of osmotic pressure on somatic embryo in *Pinus densiflora*. J. For. Res..

[48] Businge E., Bygdell J., Wingsle G., Moritz T., Egertsdotter U. (2013). The effect of carbohydrates and osmoticum on storage reserve accumulation and germination of Norway spruce somatic embryos. Physiol. Plant.

[49] Maruyama T.E., Hosoi Y., Jain S.M., Gupta P.K. (2018). Protocol for somatic embryogenesis in Japanese black pine (*Pinus thunbergii* Parl.) and Japanese red pine (*Pinus densiflora* Sieb. et Zucc.). Step Wise Protocols for Somatic Embryogenesis of Important Woody Plants Volume 1.

[50] Kermode A. (2005). Role of abscisic acid in seed dormancy. J. Plant Growth Regul..

[51] Kong L., von Aderkas P. (2007). Genotype effects on ABA consumption and somatic embryo maturation in interior spruce (*Picea glauca* x *engelmanni*). J. Exp. Bot..

[52] Rai M.K., Shekhawat N.S., Gupta A.K., Phulwaria M., Ram K., Jaiswal U. (2011). The role of abscisic acid in plant tissue culture: A review of recent progress. Plant Cell Tissue Organ Cult..

[53] Roberts D.R., Sutton B.C.S., Flinn B.S. (1990). Synchronous and high frequency germination of interior spruce somatic embryos following partial drying at high relative humidity. Can. J. Bot..

[54] Dunstan D.I., Bethune T.D., Abrams S.R. (1991). Racemic abscisic acid and abscisyl alcohol promote maturation of white spruce (*Picea glauca*) somatic embryos. Plant Sci..

[55] Montalbán I.A., Moncaleán P., Jain S.M., Gupta P. (2018). *Pinus radiata* (D. Don) somatic embryogenesis. Step Wise Protocols for Somatic Embryogenesis of Important Woody Plants.

[56] Lelu-Walter M.A., Pãques L.E. (2009). Simplified and improved somatic embryogenesis of hybrid larches (*Larix* x *eurolepis* and *Larix* × *marschlinsii*). Perspectives for breeding. Ann. For. Sci..

[57] Lambardi M., Harry I.S., Menabeni D., Thorpe T.A. (1995). Organogenesis and somatic embryogenesis in *Cupressus sempervirens*. Plant Cell Tissue Organ Cult..

[58] Bonga J.M., Von Aderkas P., Ahuja M.R., Libby W.J. (1993). Rejuvenation of tissues from mature conifers and its implication for propagation in vitro. Clonal Forestry, I., Genetics and Biotechnology.

[59] Li X.Y., Huang F.H. (1996). Induction of somatic embryogenesis in loblolly pine (*Pinus taeda* L.). Cell. Dev. Biol. Plant.

[60] Li X.Y., Huang F.H., Gbur E.E. (1997). Polyethylene glycol-promoted development of somatic embryo in loblolly pine (*Pinus taeda* L.). Cell. Dev. Biol. Plant.

[61] Carlsson J., Egertsdotter U., Ganeteg U., Svennerstam H. (2019). Nitrogen utilization during germination of somatic embryos of Norway spruce: Revealing the importance of supplied glutamine for nitrogen metabolism. Trees.

[62] Guevin T.G., Kirby E.G., Ahuja M.R., Boerjan W., Neale D. (1996). Effects of glutamine and osmoticum on somatic embryo maturation in Norway spruce (*Picea abies*) (l.) karst. Somatic Cell Genetics and Molecular Genetics of Trees.

[63] Dal Vesco L.L., Guerra M.P. (2001). The effectiveness of nitrogen sources in *Feijoa* somatic embryogenesis. Plant Cell Tissue Organ Cult..

[64] Montalbán I.A., de Diego N., Moncaleán P. (2012). Enhancing initiation and proliferation in radiata pine (*Pinus radiata* D. Don) somatic embryogenesis through seed family screening, zygotic embryo staging and media adjustments. Acta Physiol. Plant.

[65] Hargreaves C.L., Reeves C.B., Gough K., Montalbán I.A., Low C., van Ballekom S., Dungey H.S., Moncaleán P. (2017). Nurse tissue for embryo rescue: Testing new conifer somatic embryogenesis methods in a F_1_ hybrid pine. Trees.

[66] Rahmouni S., El Ansari Z.N., Badoc A., Martin P., El Kbiach M.L., Lamarti A. (2020). Effect of amino acids on secondary somatic embryogenesis of Moroccan cork oak (*Quercus suber* L.) tree. Am. J. Plant Sci..

[67] Montalbán I.A., Castander-Olarieta A., Hargreaves C., Gough K., Reeves C., van Ballekom S., Goicoa T., Ugarte M.D., Moncaleán P. (2021). Hybrid pine (*Pinus attenuata* x *Pinus radiata*) somatic embryogenesis: What do prefer, mother or nurse?. Forests.

[68] Cánovas F.M., Avila C., Cantón F.R., Cañas R.A., de la Torre F. (2007). Ammonium assimilation and amino acid metabolism in conifers. J. Exp. Bot..

[69] Llebres M.T., Pascual M.B., Debille S., Trontin J.F., Harvengt L., Avila C., Canovas F.M. (2017). The role of arginine metabolic pathway during embryogenesis and germination in maritime pine (*Pinus pinaster*). Tree Physiol..

[70] De Oliveira L.F., Navarro B.V., Cerruti G.V., Elbl P., Minocha R., Minocha S.C., Wendt dos Santos A.L., Floh E.l.S. (2018). Polyamine- and amino acid-related metabolism: The roles of arginine and ornithine are associated with the embryogenic potential. Plant Cell Physiol..

[71] Maruyama E., Hosoi Y., Ishii K. (2005). Somatic embryo production and plant regeneration of Japanese black pine (*Pinus thunbergii*). J. For. Res..

[72] Maruyama E., Hosoi Y., Ishii K. (2005). Propagation of Japanese red pine (*Pinus densiflora* Zieb. et Zucc.) via somatic embryogenesis. Prop. Ornam. Plants.

[73] Maruyama E., Hosoi Y., Ishii K. (2007). Somatic embryogenesis and plant regeration in Yakutanegoyou, *Pinus armandii* Franch. var. *amamiana* (Koidz.) Hatusima, an endemic and endangered species in Japan. Cell. Dev. Biol. Plant.

[74] Maruyama E.T., Hosoi Y., Katsuki T. (2011). Plant regeneration of Yatsugataketouhi (*Picea koyamae*) through somatic embryogenesis. Kanto Shinrin Kenkyu.

[75] Hosoi Y., Maruyama T.E. (2012). Plant regeneration from embryogenic tissue of *Pinus luchuensis* Mayr, and endemic species in Ryukyu island, Japan. Plant Biotechnol..

[76] Breton D., Harvengt L., Trontin J.F., Bouvet A., Favre J.M. (2005). High subculture frequency, maltose-based and hormone-free medium sustained early development of somatic embryos in maritime pine. Cell. Dev. Biol. Plant.

[77] Venables W.N., Ripley B.D. (2002). Modern Applied Statistics with S.

[78] Hothorn T., Bretz F., Westfall P. (2008). Simultaneous Inference in General Parametric Models. Biom. J..

[79] Stasolla C., Yeung E.C., Thorpe T.A. (2002). Maturation of somatic embryo in conifers: Morphogenesis, physiology, biochemistry, and molecular biology. Cell. Dev. Biol. Plant.

[80] Izuno A., Maruyama T.E., Ueno S., Ujino-Ihara T., Moriguchi Y. (2020). Genotype and transcriptome effects on somatic embryogenesis in *Cryptomeria japonica*. PLoS ONE.

[81] Attree S.M., Fowke L.C. (1993). Embryogeny of gymnosperms: Advances in synthetic seed technology of conifers. Plant Cell Tissue Organ Cult..

[82] Stasolla C., Yeung E.C. (2003). Recent advances in conifer somatic embryogenesis: Improving somatic embryo quality. Plant Cell Tissue Organ Cult..

[83] Texeira da Silva J.A., Malabadi R.B. (2012). Factors affecting somatic embryogenesis in conifers. J. For. Res..

[84] Nic-Can G.I., Avilez-Montalvo J.R., Aviles-Montalvo R.N., Márquez-López R.E., Mellado-Mojica E., Galaz-Ávalos R.M., Loyola-Vargas V.M., Loyola-Vargas V.M., Ochoa-Alejo N. (2016). The relationship between stress and somatic embryogenesis. Somatic Embryogenesis: Fundamental Aspects and Applications.

[85] Egertsdotter U. (2019). Plant physiological and genetical aspects of the somatic embryogenesis process in conifers. Scand. J. For. Res..

[86] Elmer K.H.S., Aslam J., Srivastava P.S., Sharma M.P. (2013). Factors regulating somatic embryogenesis in plants. Somatic Embryogenesis and Gene Expression.

[87] Gulzar B., Mujib A., Malik M.Q., Sayeed R., Mamgain J., Ejaz B. (2020). Genes, proteins and other networks regulating somatic embryogenesis in plants. J. Gen. Eng. Biotechnol..

