# Supplementary material for: Factors Influencing Somatic Embryo Maturation in Sugi (Japanese Cedar, Cryptomeria japonica (Thunb. ex L.f.) D. Don)
Source: Plants (Basel). 2021 Apr 26;10(5):874. doi: 10.3390/plants10050874 (PMC8145757; doi:10.3390/plants10050874)
Supplement: Supplementary file 1 [file plants-10-00874-s001.zip › plants-1174201-supplementary/Plants-1174201-SM-for proof.pdf]

plants

Article

Factors Influencing Somatic Embryo Maturation in Sugi [Japanese Cedar, *Cryptomeria japonica* (Thunb. ex L.f.) D. Don]

Supplementary Materials

Supplementary Tables

Table S1. Summary of the estimated parameters of the generalized linear model (GLM) of data from Table 1 [Effect of basal salts (BS) of medium on somatic embryo maturation efficiency of sugi embryogenic cell lines (ECLs)].

Variables	Estimated parameters	SE	Z value	P value	Significance
(Intercept)	5.615	0.234	23.973	0	***
S-73	-0.417	0.309	-1.352	0.176	ns
S-100	-0.363	0.309	-1.178	0.239	ns
S-113	-0.927	0.31	-2.988	0.003	**
S-352	-0.812	0.31	-2.622	0.009	**
MS	-4.198	0.401	-10.481	0	***
WP	-0.894	0.31	-2.881	0.004	**
B5	-2.235	0.321	-6.957	0	***
plate2	0.271	0.149	1.823	0.068	ns
plate3	0.299	0.149	2.01	0.044	*
S-73:MS	-0.359	0.632	-0.568	0.57	ns
S-100:MS	-0.291	0.619	-0.47	0.638	ns
S-113:MS	0.169	0.631	0.269	0.788	ns
S-352:MS	0.31	0.605	0.513	0.608	ns
S-73:WP	-0.018	0.441	-0.041	0.968	ns
S-100:WP	-2.657	0.491	-5.417	0	***
S-113:WP	-2.28	0.502	-4.542	0	***
S-352:WP	-1.814	0.475	-3.823	0	***
S-73:B5	-1.083	0.489	-2.214	0.027	*
S-100:B5	-0.858	0.479	-1.792	0.073	ns
S-113:B5	-0.434	0.485	-0.896	0.37	ns
S-352:B5	-0.38	0.479	-0.794	0.427	ns

***, $P < 0.001$; **, $P < 0.01$; *, $P < 0.05$; ns, not significant.

Table S2. Summary of the estimated parameters of the generalized linear model (GLM) of data from Table 3 [Effect of polyethylene glycol (PEG) concentration on somatic embryo maturation efficiency of sugi embryogenic cell lines (ECLs)].

Variables	Estimated parameters	SE	Z value	P value	Significance
(Intercept)	5.408	0.204	26.567	0	***
S-100	-0.312	0.275	-1.133	0.257	ns
S-352	-0.055	0.274	-0.202	0.84	ns
T-1151	0.022	0.274	0.08	0.936	ns
T-158	-0.339	0.275	-1.232	0.218	ns
PEG 15%	0.913	0.272	3.352	0.001	***
PEG 17.5%	1.069	0.272	3.925	0	***
PEG 20%	-1.792	0.288	-6.23	0	***
plate2	-0.056	0.108	-0.522	0.601	ns
plate3	0.014	0.108	0.128	0.898	ns
S-100: PEG 15%	-0.227	0.387	-0.588	0.556	ns
S-352: PEG 15%	-0.272	0.386	-0.706	0.48	ns
T-1151: PEG 15%	-0.192	0.385	-0.499	0.618	ns
T-158: PEG 15%	-0.205	0.387	-0.529	0.597	ns
S-100: PEG 17.5%	0.121	0.386	0.314	0.754	ns
S-352: PEG 17.5%	-0.31	0.386	-0.805	0.421	ns
T-1151: PEG 17.5%	0.221	0.385	0.575	0.565	ns
T-158: PEG 17.5%	0.404	0.386	1.047	0.295	ns
S-100: PEG 20%	-0.31	0.417	-0.743	0.457	ns
S-352: PEG 20%	0.396	0.404	0.981	0.327	ns
T-1151: PEG 20%	0.448	0.402	1.112	0.266	ns
T-158: PEG 20%	0.522	0.406	1.288	0.198	ns

***, $P < 0.001$; ns, not significant.

Table S3. Summary of the estimated parameters of the generalized linear model (GLM) of data from Table 5 [Effect of abscisic acid (ABA) concentration on somatic embryo maturation efficiency of sugi embryogenic cell lines (ECLs)].

Variables	Estimated parameters	SE	Z value	P value	Significance
(Intercept)	3.055	0.25	12.195	0	***
S-64	-2.255	0.518	-4.351	0	***
S-85	-2.081	0.492	-4.232	0	***
S-100	-0.805	0.374	-2.15	0.032	*
S-113	-2.423	0.547	-4.429	0	***
S-352	-1.739	0.448	-3.879	0	***
T-1151	-0.866	0.378	-2.293	0.022	*
ABA 100 μM	3.025	0.319	9.493	0	***
ABA 200 μM	2.145	0.321	6.693	0	***
ABA 50 μM	2.322	0.32	7.256	0	***
plate2	-0.194	0.106	-1.835	0.067	ns
plate3	-0.155	0.105	-1.468	0.142	ns
S-64: ABA 100 μM	2.218	0.594	3.73	0	***
S-85: ABA 100 μM	1.658	0.572	2.899	0.004	**
S-100: ABA 100 μM	0.759	0.474	1.601	0.109	ns
S-113: ABA 100 μM	1.904	0.62	3.07	0.002	**
S-352: ABA 100 μM	1.585	0.535	2.964	0.003	**
T-1151: ABA 100 μM	0.937	0.477	1.965	0.049	*
S-64: ABA 200 μM	0.272	0.607	0.448	0.654	ns
S-85: ABA 200 μM	2.822	0.573	4.929	0	***
S-100: ABA 200 μM	0.791	0.477	1.659	0.097	ns
S-113: ABA 200 μM	1.693	0.623	2.716	0.007	**
S-352:ABA 200 μM	0.673	0.54	1.245	0.213	ns
T-1151:ABA 200 μM	0.717	0.48	1.495	0.135	ns
S-64: ABA 50 μM	2.562	0.596	4.302	0	***
S-85: ABA 50 μM	2.296	0.573	4.009	0	***
S-100: ABA 50 μM	1.414	0.475	2.975	0.003	**
S-113: ABA 50 μM	0.761	0.627	1.213	0.225	ns
S-352: ABA 50 μM	0.394	0.541	0.728	0.467	ns
T-1151: ABA 50 μM	1.209	0.478	2.529	0.011	*

***, $P < 0.001$; **, $P < 0.01$; *, $P < 0.05$; ns, not significant.

Table S4. Summary of the estimated parameters of the generalized linear model (GLM) of data from Table 7 [Effect of potassium chloride (KCl) supplementation on somatic embryo maturation efficiency of sugi embryogenic cell lines (ECLs)].

Variables	Estimated parameters	SE	Z value	P value	Significance
(Intercept)	5.94	0.199	29.875	0	***
S-85	-0.261	0.248	-1.051	0.293	ns
S-100	-0.156	0.248	-0.628	0.53	ns
S-182	-0.662	0.249	-2.66	0.008	**
S-352	-0.252	0.248	-1.015	0.31	ns
T-1151	0.172	0.248	0.694	0.488	ns
T-158	0.001	0.248	0.003	0.997	ns
KCl (+)	0.098	0.248	0.398	0.691	ns
plate2	-0.012	0.148	-0.083	0.934	ns
plate3	0.097	0.148	0.653	0.514	ns
plate4	0	0.148	-0.003	0.998	ns
plate5	-0.046	0.148	-0.308	0.758	ns
S-85: KCl (+)	-0.42	0.351	-1.196	0.232	ns
S-100: KCl (+)	-0.149	0.351	-0.426	0.67	ns
S-182: KCl (+)	-0.058	0.352	-0.164	0.869	ns
S-352: KCl (+)	0.034	0.351	0.098	0.922	ns
T-1151: KCl (+)	-0.325	0.35	-0.928	0.353	ns
T-158: KCl (+)	-0.072	0.35	-0.204	0.838	ns

***, $P < 0.001$; **, $P < 0.01$; ns, not significant.

Table S5. Summary of the estimated parameters of the generalized linear model (GLM) of data from Table 9 [Effect of amino acid (AA) concentration on somatic embryo maturation efficiency of sugi embryogenic cell lines (ECLs)].

Variables	Estimate parameters	SE	Z value	P value	Significance
(Intercept)	3.734	0.238	15.657	0	***
S-100	-2.639	0.454	-5.808	0	***
S-113	-1.84	0.381	-4.827	0	***
S-182	-2.889	0.488	-5.921	0	***
S-352	-2.89	0.488	-5.921	0	***
AA 1 X	2.063	0.31	6.648	0	***
AA 2 X	2.379	0.31	7.678	0	***
AA 3 X	1.984	0.31	6.393	0	***
plate2	0.005	0.126	0.036	0.971	ns
plate3	0.004	0.126	0.032	0.975	ns
S-100: AA 1 X	1.892	0.545	3.472	0.001	***
S-113: AA 1 X	1.381	0.485	2.848	0.004	**
S-182: AA 1 X	2.543	0.573	4.442	0	***
S-352: AA 1 X	2.683	0.572	4.687	0	***
S-100: AA 2 X	2.354	0.544	4.33	0	***
S-113: AA 2 X	0.475	0.486	0.978	0.328	ns
S-182: AA 2 X	2.387	0.572	4.172	0	***
S-352: AA 2 X	2.641	0.572	4.618	0	***
S-100: AA 3 X	2.533	0.544	4.655	0	***
S-113: AA 3 X	0.665	0.487	1.366	0.172	ns
S-182: AA 3 X	2.898	0.572	5.064	0	***
S-352: AA 3 X	2.882	0.572	5.034	0	***

***, $P < 0.001$; **, $P < 0.01$; ns, not significant.

Table S6. Summary of the estimated parameters of the generalized linear model (GLM) of data from Table 11 [Effect of proliferation culture media (PCM) on somatic embryo maturation efficiency of sugi embryogenic cell lines (ECLs)].

Variable	Estimate parameters	SE	Z value	P value	Significance
(Intercept)	6.028	0.165	36.482	0	***
S-100	-0.34	0.209	-1.621	0.105	ns
T-1151	0.237	0.209	1.136	0.256	ns
T-158	0.048	0.209	0.23	0.818	ns
EM10-0	-0.341	0.209	-1.63	0.103	ns
EM3-1	-0.115	0.209	-0.551	0.582	ns
EM3-1m	-0.35	0.21	-1.672	0.095	ns
plate2	-0.107	0.117	-0.912	0.362	ns
plate3	-0.072	0.117	-0.619	0.536	ns
plate4	-0.05	0.117	-0.425	0.671	ns
plate5	-0.183	0.117	-1.559	0.119	ns
S-100: EM10-0	0.264	0.297	0.888	0.374	ns
T-1151: EM10-0	0.261	0.296	0.884	0.377	ns
T-158: EM10-0	0.054	0.296	0.183	0.855	ns
S-100:EM3-1	0.155	0.296	0.522	0.602	ns
T-1151:EM3-1	0.054	0.295	0.184	0.854	ns
T-158:EM3-1	0.109	0.296	0.368	0.713	ns
S-100:EM3-1m	0.195	0.297	0.659	0.51	ns
T-1151:EM3-1m	0.373	0.296	1.261	0.207	ns
T-158:EM3-1m	0.23	0.296	0.776	0.438	ns

***, $P < 0.001$; ns, not significant.

© 2021 by the authors. Submitted for possible open access publication under the terms and conditions of the Creative Commons Attribution (CC BY) license (http://creativecommons.org/licenses/by/4.0/).
